# Glycogen storage diseases-time to flip the outdated diagnostic approach centered on liver biopsy with the molecular testing

**DOI:** 10.12669/pjms.36.2.1310

**Published:** 2020

**Authors:** Sibtain Ahmed, Bushra Afroze

**Affiliations:** 1 Dr. Sibtain Ahmed, FCPS. Department of Pathology and Laboratory Medicine, Aga Khan University, Karachi, Pakistan; 2 Dr. Bushra Afroze, FCPS. Department of Paediatrics & Child Health, Aga Khan University, Karachi, Pakistan

**Keywords:** Glycogen Storage Diseases, Molecular Testing, Diagnostic approach

## Abstract

The glycogen storage diseases (GSDs) are a group of inherited metabolic disorders that result from a defect in any one of several enzymes required for either glycogen synthesis or glycogen degradation. The traditional diagnostic approach is based on the invasive hepatic or muscle biopsies, which are neither cost effective nor convenient. Molecular (gene testing) has emerged over the course of past few years as a robust alternative diagnostic tool, which not only confirms the diagnosis of GSDs but also clearly differentiates the types of GSDs allowing the initiation of the type-specific appropriate treatment for the particular type of GSDs. The aim of this update is to highlight the limitations of undertaking a liver biopsy for the diagnosis of GSDs; and to further describe the pros of the molecular testing for better patient centered care.

A distinct group of inherited metabolic disorders caused by a defect of the enzymes involved in glycogen synthesis, degradation, or regulation are classified under the umbrella term of glycogen storage diseases (GSDs). Several studies have reported the overall GSD incidence to be approximately 1 case per 20000-43000 live births.^1^

Glycogen is the storage form of glucose and the chief source of non-oxidative glucose disposal. It is most copiously present in the liver and muscle cells, in addition smaller amounts are present in brain, heart, kidney, adipose tissue and erythrocytes. It serves to maintain glucose homeostasis in the liver whereas provides substrates for the adenosine triphosphate (ATP) in the muscles.^2^

The GSDs can be classified on the basis of organ affected and the enzyme deficiency involved. On the basis of target organ involved GSDs are comprised of three broad groups including; hepatic type of GSDs, muscles type of GSDs and the group affecting both liver and muscles. Each group has several different types; caused by defect of a different enzyme or a transporter, which is encoded by a different gene (Table-I).

**Table-I:** Classification of GSDs and their associated enzyme deficiencies.

GSDs with Hepatic involvement		

Types	Enzymes/Transport Defect	Genes
GSD 0	Glycogen Synthase	GYS2
GSD Ia	Glucose-6-phosphotase	G6PC
GSD Ib	Glucose-6-phosphotase transporter	SLC37A4
GSD VI	Glycogen phosphorylase (liver)	PYGL
GSD IXa	Phosphorylase kinase (α subunit)	PHKA2
GSD IXb	Phosphorylase kinase (β subunit)	PHKB
GSD IXc	Phosphorylase kinase (γ subunit)	PHKG2
GSD XI	Glucose transporter-2	SLC2A2

*GSDs with Neuromuscular involvement*		

*Types*	*Enzymes/Transport Defect*	*Genes*

GSD IIa	α-1,4 glucosidase	GAA
GSD IIb	LAMP-2 protein	LAMP2
GSD V	Glycogen phosphorylase (muscle)	PYGM
GSD VII	Phosphofructokinase	PFKM
GSD IXd	Phosphorylase kinase (δ subunit)	CLAM1

*GSDs with both Hepatic & Neuromuscular involvement*		

*Types*	*Enzymes/Transport Defect*	*Genes*

GSD III	Amylo-1,6-glucosidase	AGL
GSD IV	Amylo-1,4 ◊1,6 transglucosylase	GBE1

From a clinician’s perspective the hepatic group of GSDs often has an easily recognizable clinical phenotype usually apparent as doll-like facies, failure to thrive/short stature, hepatomegaly and the biochemical features of fasting ketotic hypoglycemia, lactic academia, raised alanine transaminase with or without hypertriglyceridemia and hyper-uricemia.^3^ The muscular group on the other end presents with neuromuscular symptoms, weakness and cardiomopathy.

In Pakistan the conventional diagnostic approach towards hepatic types of GSDs includes recognition of clinical symptoms followed by biochemical work up and ultimately the more invasive liver biopsy to reveal features consistent with fatty change, nuclear hyper-glycogenation and fibrosis. Additional electron microscopy and enzyme studies on the liver tissue are required to specify the type of GSDs. Neither the electron microscopy nor the enzyme assays for GSDs are locally available, which compromises the diagnostic yield of liver biopsy for diagnosing GSD in local setting. The liver biopsy has the following limitations, some of which are specific to Pakistan:

• Controversial diagnostic accuracy for GSDs with lack of specificity based on light microscopy only.

• The procedure is invasive with serious complications including pain, hemorrhage, bile peritonitis, penetration of abdominal viscera, pneumothorax, and even death.^4^

• The high cost including multiple clinic visits, anesthesia reviews and procedure cost.

• Lack of centers with trained staff with sufficient experience for performing liver biopsy, further adds to the constraints especially for patients in smaller cities with limited technical expertise available.

• Usually 30–40 mg of tissue or four cores of hepatic tissue including about 15 mg of snap-frozen hepatic tissue in liquid nitrogen is required for all the studies necessary including light microscopy, electron microscopy and enzymes analysis to make a definitive diagnosis.^5^

• Specialized laboratories offering enzyme studies for hepatic GSDs are not available in Pakistan thus liver tissues are needed to be out-sourced to overseas laboratories in completely frozen condition with very vigilant temperature control, which is extremely challenging and chances of losing the precious hepatic tissue collected after an invasive procedure are very high.

• Electron microscopy on the hepatic tissue, which is required for the diagnosis of GSDs is not readily available at most diagnostic centers in the country owing to its high cost.

• The treatment is aimed at specific type of GSDs, which is not possible based on only histo-pathological findings of hepatic tissue on light microscopy without the enzyme testing, which is not available in Pakistan.

On the other molecular testing, which was previously only performed to supplement enzyme activity analysis and confirm equivocal results has now become the principal diagnostic tool for GSDs.^6^The distinct advantages of the molecular approach are as follows:

• Non-invasive compared to the liver biopsy.

• High diagnostic accuracy as molecular methods minimize false positive test results by targeting the specific gene of interest.

• Molecular testing clearly differentiates between different types of GSDs allowing physicians to initiate specific type-based treatment of GSDs and organizing the specific type-based surveillance plan, which varies significantly for various GSDs.

• Ease of sample transportation to the laboratory without vigilant temperature control.

• Automated analyzers are available and a single trained pathologist can report numerous samples.

• Short turnaround times, which is 48 hours.

• Cost-effective, as the cost of next-generation sequencing (NGS) allowing analysis of multiple genes in a single DNA sample is around $278.^7^ It is roughly half the cost of a liver biopsy procedure followed by light microscopy, electron microscopy and enzyme testing. Although, the liver biopsy procedure followed by light microscopy in public sector hospitals is provided free to patients, but the indirect and direct cost is actually borne by the state, which includes the liver biopsy procedure performed by a physician/pathologist, medical supplies involved in liver biopsy procedure, anesthesia coverage for the procedure, day care admission cost and the clinical care needed following an invasive procedure.

• Provision of reliable prenatal diagnosis and carrier testing by offering targeted familial variant testing to at-risk couples and carrier testing for family members.

Early diagnosis of GSDs is imperative for initiation of appropriate treatment and achieving better prognosis. Owing to the nonspecific clinical presentation of GSDs and the lack of specific biomarkers to differentiate various types of GSD, NGS has become the first line diagnostic tool for the evaluation of GSD. NGS depends on massive molecular parallelization and allows analyzing multiple genes at the same time in a single DNA sample, it is a rapid and a much cost-effective way of not only diagnosing GSD but also differentiate different types of GSDs in absence of the invasive procedure of liver biopsy.

In context of NGS instrumentation availability, a wide variety exists e.g. MiniSeq and Miseq from Illumina, Ion Torrent from Life Technologies Thermo Fischer Scientific and MinION from Oxford Nanopore etc. These differ primarily in terms of cost, capacity, principle chemistry and read length, DNA library preparation and run time. On the basis of feasibility, the Illumina MiSeq is the most popular sequencer due to its relatively low cost, significantly low error rate and capacity to deliver the moderate throughput required by most centers.^7^

From a local perspective, measurement of enzyme activity suffers from logistic issues and is technically more challenging alongside a dearth of laboratory expertise. Furthermore, enzyme analysis is usually not reliable in detecting heterozygous carriers of a disease. These testing modalities are often very laborious, time consuming and require a pre-selection by clinical phenotype for targeting the specific enzyme.^8^ On the contrary, NGS has now become the gold standard to confirm a suspected diagnosis of GSD owing to its comparatively low cost, rapid analysis time and availability of less technically demanding automated platforms. Enzyme analysis on hepatic tissue is only needed in certain cases when there are unclear molecular results like variants of uncertain significance found on gene testing.

In line with the best practices, the undertaking of molecular testing for suspected cases with GSDs, by outsourcing the samples abroad to accredited laboratories has been a standard practice at our institute. For this purpose, the extracted DNA samples from peripheral (whole) blood specimen is outsourced for NGS based GSDs panels, with ease of transportation at room temperature.

The capacity and capability to perform molecular testing using NGS is available in Pakistan. NGS equipment and proficient molecular pathologists and molecular geneticists are present at few centers locally, but molecular testing for GSDs is currently not offered in the country. In most clinical settings, the pediatricians and pediatric metabolic geneticists outsource NGS molecular testing to various accredited centers abroad, offering GSDs panels. However, the expertise of local molecular pathologists and molecular geneticists can be easily nurtured in this respect; and the widespread use of molecular testing of GSD can be achieved with its added distinct advantages.

## Authors’ Contributions:

**BA** conceived, designed the idea and did final review of the manuscript, is responsible for integrity of research.

**SA** did literature review and manuscript writing.
